# Stereotypical Behaviors in Chimpanzees Rescued from the African Bushmeat and Pet Trade

**DOI:** 10.3390/bs3010001

**Published:** 2012-12-27

**Authors:** Stacy M. Lopresti-Goodman, Marjanne Kameka, Ashlynn Dube

**Affiliations:** Marymount University, Department of Psychology, 2807 North Glebe Road, Arlington, VA 22207, USA; E-Mails: Marjanne_Kameka@Marymount.edu (M.K.); Ashlynn_Dube@Marymount.edu (A.D.)

**Keywords:** chimpanzees, abnormal behaviors, stereotypical behaviors, post-traumatic stress disorder, bushmeat

## Abstract

Many orphaned chimpanzees whose mothers are illegally killed for their meat (bushmeat) in Africa are sold as pets or kept caged at hotels and businesses to attract tourists. As a result of being separated from their mothers and other chimpanzees at an early age, and spending years in impoverished captive conditions, some of these individuals engage in abnormal behaviors, including stereotypically scratching at their flesh and repetitively rocking back and forth. This paper presents case studies of Poco and Safari, two chimpanzees who were rescued by sanctuaries after living alone on display for humans at businesses for the first 7 to 8 years of their lives. Decades after their rescue, they still engage in stereotypical behaviors as a result of the psychological and physical trauma they endured early on. This paper combines data from in depth interviews with caregivers and direct observations of abnormal behaviors to assess psychological distress in captive-living chimpanzees. Our results highlight some lesser known harms of the bushmeat trade and the detrimental life-long consequences that keeping chimpanzees as “pets” can have on their mental health.

## 1. Introduction

Over the past decade, the illegal hunting of chimpanzees in Central and Equatorial Africa for their meat (bushmeat) has been on the rise [1,2,3]. Not only does this introduce risks for zoonotic disease transmission, but chimpanzee hunting results in an increased number of orphaned chimpanzees being sold as “pets” [4]. Many of these orphans die as a result of the trauma they endure during capture or from illness, malnutrition, neglect or physical abuse they endure while being kept as pets [1,3,5]. 

Many chimpanzees who have been rescued from the bushmeat or pet trades have experienced early maternal separation and have lived in restrictive captive environments lacking social or cognitive stimulation. In the wild, chimpanzees spend the first 5 years of their lives nearly inseparable from their mothers [6,7,8]. Similar to what happens in humans and other non-human primates, maternal deprivation and social isolation at an early age can result in chimpanzees developing a variety of abnormal behaviors that can persist in perpetuity [6,8,9,10,11,12,13,14,15,16,17,18]. Typically, behaviors are deemed “abnormal” if they are species atypical or occur only or more often in captive chimpanzees [6,18]. 

Some of the most common abnormal behaviors seen in captive-living chimpanzees include coprophagy (eating of feces), regurgitation and reingesting (R&R) of food, rocking back and forth, and self-clasping [9,17,18,19,20]. Additionally, these chimpanzees may engage in auto-aggressive behaviors, such as self-hitting, self-biting, pulling out their own hair, and scratching into their own flesh, or may engage in stereotypic behavior, frequently performed behaviors that have little variation in form and appear to serve no obvious function [6,9,15,18,21]. For example, they may engage in repetitive self-grooming in a localized area to the point they make themselves bald. While such behaviors may appear functionless, it is possible that these behaviors are engaged in as a means of self-stimulation in the absence of adequate enrichment, or could be performed as a means of coping with stress [6,9,12,20,22,23,24,25]. In traumatized humans, self-harm may be attributed as an outward physical manifestation of internal psychological distress [26]. In addition to exhibiting abnormal or stereotypic behaviors, as seen in other primates who were restrictively reared, rescued chimpanzees often have difficulties socializing with conspecifics once reintroduced into a chimpanzee population [6,14,15,16,18,27,28,29,16,18,27]. This could result in them having reduced levels of play and grooming and having absent or abnormal sexual behaviors.

Some orphans are fortunate enough to be rescued by accredited sanctuaries whose goal is to rehabilitate and provide refuge for the orphaned chimpanzees in socially rich and physically complex environments [1]. Recent research conducted at one African sanctuary demonstrated that a majority of chimpanzees living there do not exhibit abnormal behaviors, despite any possible negative early life experiences [20]. The authors proposed that this was most likely the result of the orphans being rescued at an early age, and subsequently peer-raised with complex environmental stimulation. They suggest this compensated for any early deprivation the orphan chimpanzees may have experienced. Other researchers have found, however, that many abnormal behaviors and signs of psychological distress are not reversible, and may persist for decades after cessation of deprivation [10,13,14,16]. While a majority of all chimpanzees rescued by African sanctuaries are below the age of 4, there is still a large proportion—according to one estimate, approximately 20%—who are not rescued until they are much older [1]. These individuals most likely experienced prolonged years of social isolation before being rescued. Despite living in enriched sanctuary environments with conspecifics for decades, some of these chimpanzees continue to show signs of psychological distress. 

It has been proposed that chimpanzees engaging in abnormal or stereotypic behaviors might be indicative of psychopathology similar to that in humans [6,12]. Given homologies in brain structures affected by distress in human and non-human animals (e.g., the hippocampus, the hypothalamic-pituitary-adrenal axis), and psychosocial and behavioral similarities between humans and chimpanzees [6,21], researchers have applied the criteria from the *Diagnostic and Statistical Manual of Mental Disorders, 4^th^ Ed.* (*DSM-IV*) which is used to diagnose humans with psychopathologies such as depression or Post-Traumatic Stress Disorder (PTSD), to chimpanzees [12,30,31,32,33]. 

According to the *DSM-IV*, PTSD is an anxiety disorder that occurs after one has seen or experienced a traumatic event that involved the threat of injury or death, such as being in a severe car accident [30]. Symptoms include intrusive recollection, characterized by becoming emotionally and physically upset by reminders of the traumatic event, avoiding certain places, people or activities, exhibiting social withdrawal, having excessive outbursts, being easily made anxious or startled, and always being on guard (hypervigilance). Complex PTSD (CPTSD) is a form of PTSD that occurs as a result of the early onset of trauma, and with prolonged and repetitive trauma. This might occur when the victim is held captive and under the control of a perpetrator [34], as is the case with prisoners of war, hostage survivors, or victims of physical domestic abuse [26,34,35,36]. Both PTSD and CPTSD include the source of the trauma as part of the definition of the disorder [12,30]. Similar to these harmful situations humans may encounter, chimpanzees who are kept as “pets” or used in biomedical research experience traumatic confinement conditions and oppressive relationships that result in CPTSD-like symptoms [12]. Beyond the symptoms for PTSD listed above, symptoms of CPTSD may also include affect dysregulation, automatization of behavior, dissociative coping, and somatization, such as overreacting to minor stressors. Individuals who exhibit symptoms of CPTSD have been found to have increased usage of self-injury as a way of self-soothing [37]. 

Diagnosing psychopathologies in humans is done via qualitative assessments by a clinician. If the patient reports a specific number and kind of symptoms outlined in the *DSM-IV* for a specific disorder, then they are said to meet the criteria for that disorder. Since chimpanzees cannot give verbal reports of their symptoms, researchers have relied upon qualitative reports from their human caregivers. While some may dismiss a chimpanzees’ lack of the ability to verbalize the source of trauma or symptoms as a reason for why they cannot be diagnosed with psychopathologies, and suggest any attempt at doing so is anthropomorphism, others have argued that this is just as valid as a method as relying upon a human caregiver for reports of symptoms in a non-verbal human with a disorder, such as a small child [11,38]. Using this methodology, both PTSD- and CPTSD-like symptoms have been reported in chimpanzees who have experienced maternal deprivation, physical and psychological abuse, or who lived in deprived captive conditions [12,31]. 

Not all of the criteria for PTSD outlined in the *DSM-IV*, however, map neatly from humans to chimpanzees. Ferdowsian *et al*. [31] attempted to control for this by introducing a set of alternative criteria that are behaviorally anchored for use with chimpanzees and that eliminated symptoms that a human caregiver could not determine from their interactions with the chimpanzees. Similar adaptations for interspecies differences have been made to other human-based psychological assessments, such as the Big 5 Personality Inventory [39,40,41]. This is a divisive issue in the literature. While some question the validity of modifying any human-based criteria to be used on non-human animals, others have argued that we should use the same theories and models to discuss psychology of humans and other animals given the continuities between brain and behavior [6,42,43,44,45,46]. 

The goal of this paper was to combine interview data about PTSD- and CPTSD-like symptoms in chimpanzees with direct observations of abnormal and stereotypical behaviors. We hypothesized that chimpanzees who experienced prolonged confinement and separation would exhibit symptoms of PTSD and CPTSD. As a result, we expected that they would also spend a large percentage of time engaging in a variety of abnormal behaviors and would be socially withdrawn. 

## 2. Experimental Section

### 2.1. Research Site

This research was conducted at Sweetwaters Chimpanzee Sanctuary in Nanyuki, Kenya. The sanctuary is a member of the Pan-African Sanctuary Alliance (PASA), an alliance of 18 sanctuaries in 12 African countries that currently care for orphaned and/or confiscated chimpanzees. The 42 chimpanzees under Sweetwaters’ care live in two separate multi-male, multi-female mixed age groups on 250 acres of land separated by a river and protected by an electrical fence within the Ol Pejeta Nature Conservancy. Poco and Safari, the subjects of this case study, live in a group consisting of 21 other individuals (see Table 1) living on 98 acres of land. Seventeen are suspected orphans of the bushmeat trade and were rescued from individual’s homes or businesses, were confiscated from smugglers, or were surrendered as ex-pets. Three of the females and one male were born at the sanctuary. Ol Pejeta has a semi-arid climate and the chimpanzee’s habitat consists of Acacia mixed woodland and Euclea bushes. 

**Table 1:** Composition of Poco and Safari’s Group.

Age Category			
*Sex*	Infants and Juveniles	Adolescents and Young Adults	Prime, Mature, and Old Adults
	1–7 years	8–15 years	16 years +
*Males*	-	5	4
*Females*	2	3	7

The chimpanzees are fed a varied diet of fresh fruits and vegetables daily, and are also given opportunities to forage for peanuts. Within their outdoor enclosure, they have a variety of manmade climbing structures in addition to large acacia trees. The two groups sleep in two different indoor enclosures that have elevated beds for making nests in clean straw and have windows and sky lights to let in natural light. 

### 2.2. Caregiver Interviews

Six senior caregivers at Sweetwaters Chimpanzee Sanctuary volunteered to participate in the interview portion of our study. They have worked at Sweetwaters an average of 223.5 months (SD = 11.5). All procedures were approved by the Sweetwaters Chimpanzee Sanctuary board of directors, the Kenyan Ministry of Education, and Marymount University’s Institutional Review Board. 

The first stage of our project involved the first author conducting open-ended interviews with the caregivers to narrow down the pool of potential chimpanzees to focus on. The caregivers interviewed had read Walsh *et al*. [18] for a previous study they participated in [31] and were therefore familiar with definitions of the most common abnormal behaviors identified in captive-living chimpanzees. 

During the interviews, the first author asked the caregivers to list the names of any individual chimpanzees they have seen exhibit abnormal behaviors, as well as to list the different types of abnormal behaviors they had witnessed them engage in. Thirty-five of the 42 chimpanzees at the sanctuary were mentioned as exhibiting at least one abnormal behavior by at least one caregiver (approximately 83%). For those 35 chimpanzees, we determined how many different caregivers mentioned each of them (Range = 1–5; M = 2.31, SD = 1.37). We combined the lists of reported abnormal behaviors from the different caregivers and calculated the number of unique abnormal behaviors mentioned for each individual chimpanzees (Range = 1–10). To calculate an “abnormality” score for each chimpanzee, we multiplied the number of caregivers who mentioned each chimpanzee by the unique number of abnormal behaviors mentioned. We selected the five most “abnormal” chimpanzees to conduct follow-up interviews on. 

The follow-up interviews were conducted by the first author with the same six senior caregivers initially interviewed. For each chimpanzee, the two caregivers who worked most frequently with them were interviewed individually. This was an attempt to assess consistency in perceived symptoms in each chimpanzee between their two primary caregivers. During these interviews, the caregivers were asked questions based upon the alternative criteria for PTSD outlined in Ferdowsian *et al*. [31] as well as questions about whether they have seen the chimpanzee perform specific abnormal behaviors that might indicate psychological distress (coprophagy, R&R, rocking, self-clasping, self-hitting, pulling out of the hair, or scratching into their own flesh) [9,18]. 

Our interviews revealed that none of the caregivers could speak to criterion D1 of Ferdowsian *et al.* [31] relating to whether the chimpanzees had difficulty sleeping through the night, nor could they speak to criterion D3, the chimpanzees’ ability to pay attention to tasks, since we had no operational definition of “task” to assess. For the purpose of this study, those criteria were not assessed. Given an individual only needs to meet a certain number of criteria in each of the symptom clusters, however, this did not impact our ability to determine whether the chimpanzees met the alternative criteria for PTSD. Since the research reported in this paper was first conducted, Rosati *et al*. [43] have published a response to the Ferdowsian *et al*. [31] study also pointing out the difficulties of assessing these specific criteria. 

The results of our caregiver interviews revealed that three of the five chimpanzees met all 11 of the alternative criteria items for PTSD we assessed [31], and there was 100% agreement in ratings between two separate caregiver interviews. Two of those chimpanzees will be discussed in this paper, Poco and Safari.

### 2.3. Chimpanzee Background Information from Interviews

According to our caregiver interviews and inspection of the sparse information available to us in the chimpanzees’ very limited records, Poco and Safari have similar histories of trauma that likely resulted in their developing symptoms of PTSD and CPTSD. They are both orphans of the African bushmeat trade who experienced maternal separation and deprivation, as well as social isolation and stressful living conditions during the first years of their life while they were kept on display for humans. 

#### 2.3.1. Poco Background Information

**Figure 1:**
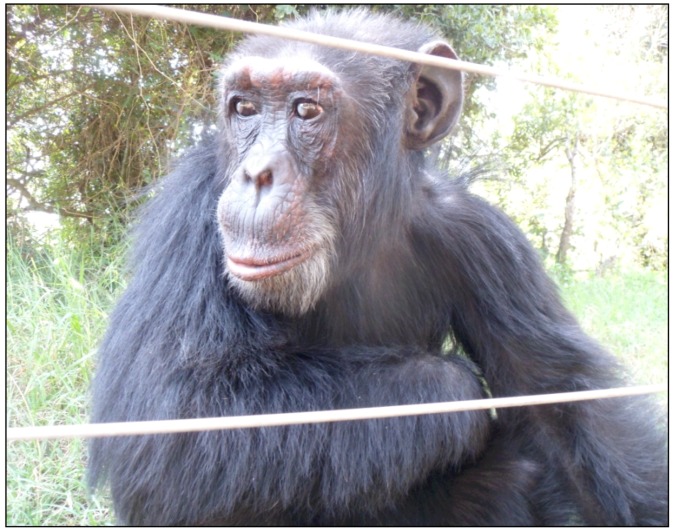
Poco at Sweetwaters Chimpanzee Sanctuary in Kenya in 2011.

**Figure 2:**
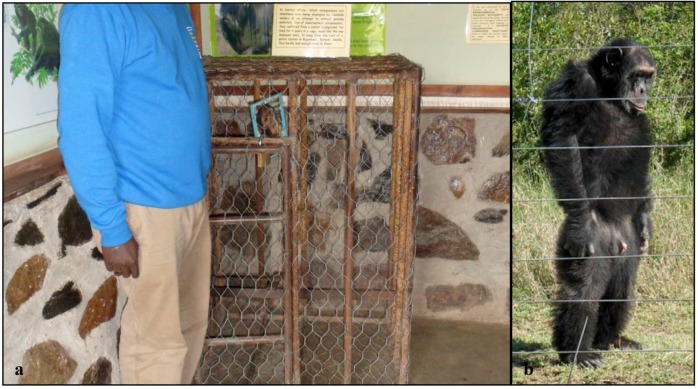
**(a)** A cage similar to the one that Poco lived in at the service station in Burundi until he was rescued in 1989; **(b)** Poco spends most of his time walking upright and bipedally as a result of the damage incurred to his back from living in such a tiny cage.

Poco (Figure 1) is estimated to have been born in 1981. It is not known how old he was when he was first separated from his mother and sold to humans, but he lived in a service station in Burudi where he was used to attract customers until he was rescued in 1989. While there, he lived alone and was kept suspended from the ceiling in a cage that was too small for him to lay down in (See Figure 2a) [47]. This resulted in him having permanent back damage which causes him to walk bipedally (Figure 2b). When he first arrived at the Jane Goodall Institute sanctuary in Bujumbura, Burundi in 1989, it is reported that he was weak and malnourished. After some time passed, he is reported to have adjusted and was considered the alpha male of his group for a period [47]. In 1995, Burundi broke out into civil war and some of the chimpanzees living at the Jane Goodall Institute sanctuary were brought to the Sweetwaters Chimpanzee Sanctuary, including Poco and Safari. 

At Sweetwaters, Poco is considered a solitary chimpanzee and prefers the company of humans to other chimpanzees. He is often found close to the observation tower, where visitors are allowed to view the chimpanzees for 3 hours a day, or is found close to the house the chimpanzees sleep in at night since this is where the caregivers spend much of their shifts. He has never been seen mating or attempting to mate with another chimpanzee but has been seen masturbating towards humans. 

Poco is characterized by caregivers as being a nervous chimpanzee who is easily startled by noises and always appears on guard (hypervigilance). He is reportedly afraid of people in uniforms and brightly colored clothing, and will try to escape and injure anyone wearing them. He is also afraid of guns or items that resemble a gun and paces or throws rocks and sticks at anyone holding anything resembling these items (intrusive recollection). He once escaped from his enclosure and attacked a wildlife security officer who was dressed in uniform and carrying a gun while patrolling the fence perimeter. Caregivers stated that have seen him self-clasp, rock, fear grimace or display in the presence of humans, and that he also will flip his upper lip up to expose his teeth to them when distressed. He is reported to sometimes seem unaware of the other chimpanzees and what they are doing around him as if he is having a dissociative episode. Caregivers say he spends a large proportion of his time poking his flesh with sticks or very sharp acacia thorns; he does not break the skin when doing this.

#### 2.3.2. Safari Background Information

**Figure 3:**
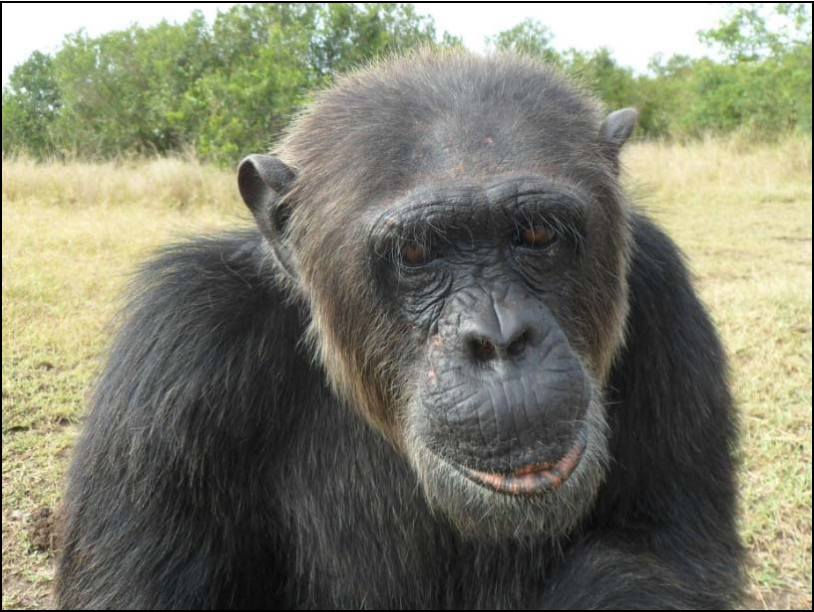
Safari at Sweetwaters Chimpanzee Sanctuary in 2011.

Safari (Figure 3) has a similar background as Poco. He is estimated to have been born in 1984. Again, the age at which he was first separated from conspecifics and sold to humans is not known. When he was rescued in 1991, however, he was being singly housed and living in a small, dark cage behind a hotel in Burundi and was used to attract tourists [47]. When he was first brought to the Jane Goodall Institute sanctuary in 1991, his behavior was labeled “neurotic and frantic”, and although he was the largest chimpanzee in his group, it was reported that he was low ranking [47].

When Safari first arrived at Sweetwaters in 1995, he was reported to have been a very aggressive and “neurotic” chimpanzee. Slowly, his aggression and neuroticism subsided. Now, Safari is a solitary chimpanzee and he too is often found poking his flesh with sticks or sharp acacia thorns, which can cause serious injury. Unlike Poco, he has pushed so hard with a thorn that he injured the skin under his chin. He has also been seen biting himself. He is said to become easily excited by exposed human skin and will often masturbate and rub his nipples at the sight of it or at the sight of human females. He has never been seen mating or attempting to mate with a chimpanzee. 

Safari is also characterized as being an anxious, easily agitated, and an easily angered chimpanzee. He gets upset at the sight of militaristic looking uniforms, gumboots, and items that resemble a gun, such as brooms (intrusive recollection). When seen, he displays physical symptoms of distress (e.g., piloerection, fear grimacing, displaying and running away), will become very aggressive, and will attempt to break out of his enclosure to get to these items or the people with these items. He is also very easily startled (hypervigilance) and made upset by flying birds and passing planes, and is afraid of sudden noises. When these objects are seen or heard, he will become physically and emotionally upset, will display and run into the bush, or will sit, self-clasp and rock back and forth. 

### 2.4. Chimpanzee Observations

The first author conducted a total of 11 hours of observation on Poco and 11 hours of observation on Safari spread out across 7 days in June of 2012. These observation times are comparable to, and in some cases greater than, the amount used in other investigations of abnormal behavior in primates [16,20,48]. For each chimpanzee, 5½ h of observations were conducted in the morning between the hours of 7:30 am, when they are first let out of the house and receive breakfast, and 12:00 pm, when they receive their lunch. The other 5½ h were collected between 12:00 pm and 5:00 pm, the time they return to their house for dinner and to go inside for the night. Poco and Safari were observed from an observation deck that abuts their enclosure or from the fence perimeter surrounding their enclosure at a distance ranging from 1.5 m to approximately 30 m. No observations were made near the visitor platform on the other side of their enclosure. They were observed in 30 min blocks using instantaneous focal animal sampling with 1 minute intervals. Missed or “bad” observations were coded as such. If either Poco or Safari were out of sight for more than 10 continuous observations, resulting in 1/3 of a session of missed or bad observations, the session was terminated and a new data collection session began when he was back in view [49,50]. The distribution of the 30 min data collection sessions was quasi-randomly distributed and depended upon who was observable at a given time. Since the chimpanzees are given free access to their large enclosures, there were times when Poco and Safari were unobservable for hours. If one reappeared, and he was not already observed for 2 h during that day, a data collection session began. Each session was separated by a minimum of 5 min and no more than 2 h of data were recorded during one day.

The first author recorded both normal and abnormal behaviors using an ethogram that was a combination of behaviors documented in other captive-living chimpanzees [9,18,51,52] but also included one novel behavior that the first author observed while conducting preliminary observations at Sweetwaters in August of 2011. This was a repetitive and sustained poking into the skin with a sharp stick or thorn. The observations were made with or without the use of binoculars. The data was recorded with pen and paper and a Sper Scientific Ltd continuous alarm timer. In addition to recording the behaviors Poco and Safari engaged in, the social context in which the behavior was occurring in (e.g., was the chimpanzee solitary, affiliating with a partner or the social group [e.g., sitting while touching, sitting within 3 m of], interacting with a partner [e.g., grooming, playing], or orienting their behavior towards humans) was recorded [51]. 

We were interested in determining the diversity of abnormal behaviors Poco and Safari performed (e.g., the number of different kinds), the percentage of observation time engaged in these abnormal behaviors, and the percentage of time spent in the different behavioral contexts [9,51]. We defined behaviors as abnormal if the behaviors are species atypical and occur only, or more often, in captive-living chimpanzees [6,18]. We hypothesized that since the caregiver interviews revealed that they exhibited many of the symptoms of PTSD and CPTSD, and experienced prolonged years of social isolation before being rescued by the sanctuary, that our direct observations would reveal that they would spend a large percentage of their time engaging in a variety of abnormal behaviors, would display the symptoms of PTSD and CPTSD their caregivers mentioned them exhibiting, and would be socially withdrawn or human oriented more often than they would be with their group.

## 3. Results and Discussion

### 3.1. Poco Naturalistic Observation Results

**Figure 4:**
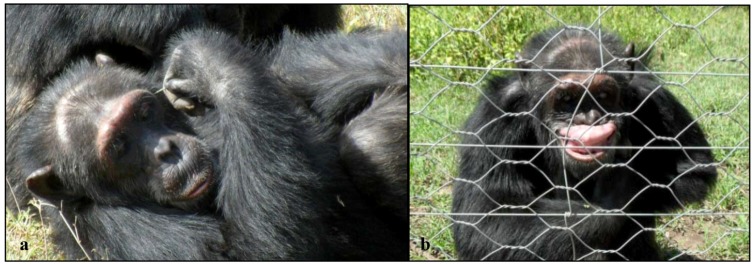
**(a)** Poco sticking a sharp acacia thorn into the side of his face; **(b)** Poco flipping his upper lip at tourists observing him from the Visitor Center at Sweetwaters.

Direct observations revealed that the diversity of abnormal behaviors that Poco engaged in numbered seven and included poking himself with a sharp thorn or stick (Figure 4a), rocking back and forth, self-clasping, grooming stereotypically, displaying in response to the arrival of humans, clapping and extending his hand in the first author’s direction, and flipping his upper lip exposing his teeth and gums (Figure 4b). Poco was engaging in these abnormal behaviors on 34% of the behavioral observations (See Table 2). The abnormal behavior that he engaged in most frequently was poking himself with a sharp stick or thorn, which he was observed doing 26% of the overall observation time. Poco was observed poking himself on his chin, ribs, neck, face, and back, and even poked himself while getting groomed by a female chimpanzee, JoJo.

**Table 2:** Poco’s Distribution of Normal and Abnormal Behaviors in Each Behavioral Context.

Behavioral Context				
*Behavior*	Human Oriented (19%)	Solitary (59%)	Affiliating (14%)	Interacting (8%)
*Normal 418 (66%)*	80 (65)	295 (79)	30 (35)	13 (25)
*Abnormal 219 (34%)*	43 (35)	80 (21)	56 (65)	40 (75)
Total N = 637*	123	375	86	53

Note: * Twenty-three of the 660 total observations were coded as “missed” observations and therefore did not factor into the percentage of time spent in each behavioral context. Data within the table are represented as N Observations (% of Observations in that Behavioral Context), while the category labels indicate (% of Total Observations).

Table 2 illustrates the percentage of each behavioral context that Poco was engaging in either normal (e.g., eating, resting, grooming, playing) or the abnormal behaviors listed above. On some occasions when he was human oriented, it appeared that the presence of humans upset him and he rocked (10 observations) in response. If he was rocking while looking at the first author, she would walk away or reposition herself such that she was not causing him to be distressed. Some instances when she did walk away, he would then clap in her direction and extend his hand to her and nod his head in a way that suggested he wanted her to return. A majority of the human-oriented behaviors, however, were him just sitting and looking at humans. Lastly, Poco was observed to be solitary on 59% of the observations, during which he often poked himself while sitting or lying down. In some instances he chose to be solitary while group members sat together on a large climbing structure, but many other times the group was off in the bush while he sat near his house or the fence. 

**Figure 5:**
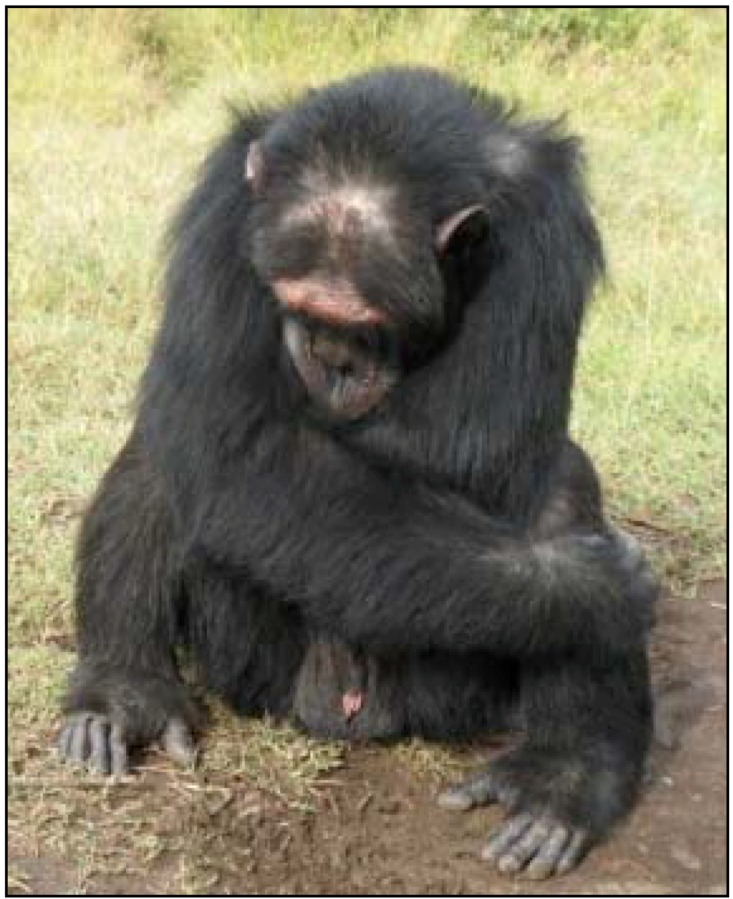
Poco sitting in a depressed, hunched posture.

Beyond the behaviors recorded and outlined above, Poco was often observed sitting in a depressed hunched posture; while self-clasping, his head often hung below his shoulders (Figure 5). He also exhibited many of the PTSD and CPTSD symptoms his caregivers mentioned such as appearing tense and constantly surveying the situation around him and he was never relaxed (hypervigilance). Relative to the other chimpanzees in his group, he appeared to overreact to distant noises and the arrival of humans (somatization, affect dysregulation). Despite once being the reported alpha of his group, Poco was very submissive and avoided all interactions with the current alpha male of the group. 

### 3.2. Safari Naturalistic Observation Results

**Figure 6:**
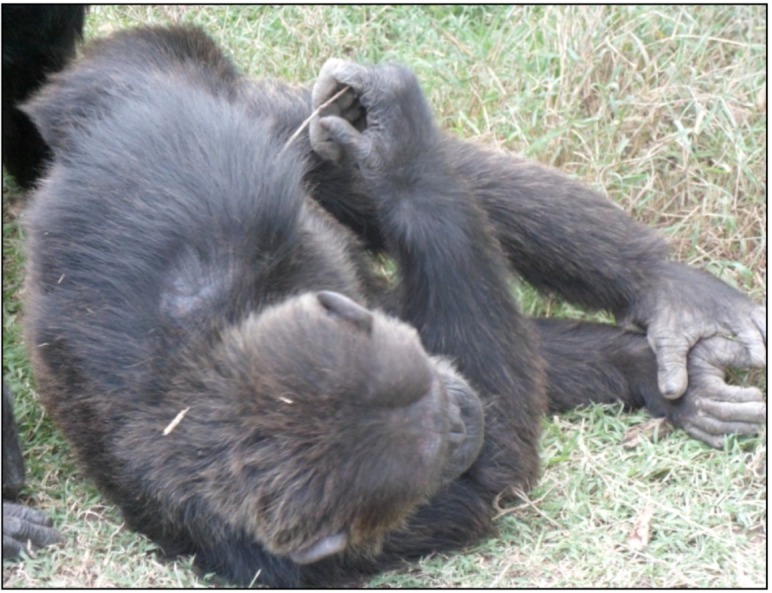
Safari poking himself with a stick in the arm.

The diversity of abnormal behaviors that Safari performed was five and included flipping his lip, rocking, hand-wiping, masturbating to humans, and poking himself with an object (Figure 6). Safari was observed engaging in these abnormal behaviors on 57% of the observations. Poking with a thorn or stick was the most frequently engaged in abnormal behavior and he was observed doing this on 332 observations, or 51% of the time. Safari poked himself on his chin, cheek, top of his head, his ear, neck, ribs, forearm, wrist, and his hand.

Safari is much more social than Poco and was observed affiliating with other chimpanzees on 28% of the observations and interacting with other chimpanzees on 16.5%. Safari engaged in his poking behavior a majority of the time he was affiliating or interacting with other chimpanzees, however, meaning he was engaging in an abnormal behavior even while engaging in a normal chimpanzee behavior. This is what resulted in him having a large number of “abnormal” behavior codes while affiliating or interacting with a partner, as detailed in Table 3.

**Table 3:** Safari’s Distribution of Normal and Abnormal Behaviors in Each Behavioral Context.

Behavioral Context				
*Behavior*	Human Oriented (9.5%)	Solitary (46%)	Affiliating (28%)	Interacting (16.5%)
*Normal 280 (43%)*	23 (37)	163 (55)	56 (31)	38 (36)
*Abnormal 368 (57%)*	39 (63)	134 (45)	126 (69)	69 (64)
Total N = 648 *	62	297	182	77

Note: * Twelve of the 660 total observations were coded as “missed” observations and therefore did not factor into the percentage of time spent in each behavioral context. Data within the table are represented as N Observations (% of Observations in that Behavioral Context), while the category labels indicate (% of Total Observations).

Safari is much less human-oriented than Poco and was only observed directing his behaviors towards humans on 9.5% of the observations. It was those observations, however, that he was masturbating, rocking, or flipping his lip. It appeared that he masturbated in response to his male caregiver having the top button of his shirt undone, however there is no way we can be certain of the exact cause. The largest percentage of observations was recorded while Safari was solitary, or 46% of the observation time (See Table 3).

### 3.3. Discussion

The purpose of this paper was to present case studies of Poco and Safari, two chimpanzees who are orphans of the African bushmeat trade and were kept as “pets” before being rescued by a sanctuary. As a result of trauma associated with maternal separation and prolonged social isolation, both display a variety of symptoms of psychological distress similar to those of PTSD and CPTSD in humans. Additionally, they engage in abnormal, stereotypical behaviors even decades after being rescued.

Like many chimpanzees and humans who experience early-life trauma and repetitive stress, both Poco and Safari were reported by their primary caregivers as displaying a constellation of symptoms that resemble those of PTSD and CPTSD in humans. Some of the symptoms reported include social withdrawal, lack of interest in interacting with other individuals (e.g., grooming or playing), physical and emotional reactions to what might be reminders of traumatic events in their past (e.g., militaristic uniforms, guns), hypervigilance, and being easily startled or scared. Some of these behaviors were observed by the first author, including social withdrawal, hypervigiliance, and extreme physical and emotional reactions to noise and the presence of humans as evidenced by their screaming, piloerection, displaying, and subsequent running into the bush. Both Poco and Safari were socially withdrawn for the majority of the observations and were recorded spending approximately 53% of the observation time alone. One weakness of this study is that we did not conduct observations of their entire group so we could compare the amount of time others were solitary to the time Poco and Safari were solitary. This was not possible for practical reasons. As soon as the chimpanzees could go outside in the morning, the majority of them went deep into the bush where they were unobservable by the first author, while Poco and Safari always stayed near the perimeter of the fence and near the house. When the rest of the chimpanzees were near the house, they were interacting as a group for the most part. 

In addition to being socially withdrawn, both individuals spent a large proportion of their time near the perimeter of the fence, or near their enclosure where the caregivers spend their days. Despite being able to go off into their forested enclosure with their group members, Poco and Safari chose to be near humans even though it appeared that the presence of humans sometimes caused them distress, as evidenced by their engaging in abnormal behaviors (e.g., lip flipping, rocking, poking). We acknowledge that this preference for staying near humans may have skewed the results and is what contributed to the high percentages of abnormal behavior; however, other investigations of abnormal behaviors in chimpanzees have found similarly high distributions [16]. 

Like human survivors of trauma who develop paradoxical positive feelings towards their captors or those who inflict abuse and domination on them, known as Stockholm syndrome, it seems that both Poco and Safari prefer to be near humans, or alone, as opposed to being with their conspecifics [35]. We must acknowledge the possibility that some of the symptoms of distress they still display are—despite the relative autonomy they now have and their rich social environment—a result of continuing to live in captivity and interacting with humans following their traumatic history with human abusers. Unfortunately, releasing Poco, Safari, and the other chimpanzees back into the wild is not an option given their inability to fend for themselves after living in captivity and being dependent upon humans for prolonged periods of time. Because most of the chimpanzees at Sweetwaters do not exhibit CPTSD-like symptoms, and previous research has found that most chimpanzees living in PASA sanctuaries do not engage in abnormal behaviors, we suggest that the abnormal behaviors reported and observed in Poco and Safari are likely the result of traumatic negative experiences prior to being rescued by the sanctuary [20]. Similarly, other researchers have concluded that abnormal behaviors that persist in chimpanzees who live in rich social environments are most likely the result of past negative experiences [16]. 

In addition to exhibiting PTSD- and CPTSD-like symptoms as reported by their caregivers, both Poco and Safari engaged in a variety of abnormal behaviors that were initially reported by their caregivers and later observed and recorded by the first author. It is important to note that some of these abnormal behaviors typically are indicative of distress, such as such as self-clasping, rocking back and forth, and flipping their lip, while others are not. Although chimpanzees in the wild have been documenting flipping their upper lip to expose their teeth and gums, this is considered a common abnormal behavior observed in captive-living chimpanzees [9,18]. We concluded that this and the other abnormal behaviors listed above are stress induced behaviors and not learned from the other chimpanzees since, anecdotally, none of the other chimpanzees in their social group were observed performing these behaviors. Further, behaviors such as rocking, self-clasping and lip flipping appeared to be performed in response to humans’ presence, indicating their discomfort with humans. 

Other abnormal behaviors, such as Safari’s masturbating towards humans, and Poco’s hand clapping to get the first author’s attention, are considered abnormal because they are species atypical behaviors that occur only or more often in captive chimpanzees; however, they most likely do not indicate distress [6,18]. For these behaviors, we must take into consideration the circumstances in which they found themselves living for the first decade of their lives. They were isolated from conspecifics and raised by humans. As seen in other chimpanzees raised in captivity for prolonged periods of time, they do not know how to engage in normal chimpanzee sexual behaviors and have never been seen mating. While masturbating has been seen in wild chimpanzee populations on rare occasions, this is most likely a behavior that they learned while living with humans or something they discovered themselves while living alone to relieve any sexual urges they do not know how to relieve otherwise. With regard to Poco’s clapping to get the first author’s attention, while this behavior is abnormal for wild chimpanzees, is not abnormal for captive-living chimpanzees who learn that this is one way to get the attention of humans. 

For both Poco and Safari, the abnormal behavior they were observed engaging in most often was poking at their flesh with a sharp thorn or stick; they were recorded doing this 26% and 51% of the observation time, respectively. This behavior can be classified as stereotypical because it is an often repeated behavior that appears to serve no function or purpose [6]. For example, it did not appear that either of them was using the thorn or stick to groom with. While it was not apparent from the first author’s vantage point that they ever broke the skin, this behavior is similar to self-injurious behaviors, such as pulling out of the hair or self-biting, which are commonly used as indicators of stress or anxiety. 

Previous investigators of self-injurious behaviors in primates have proposed these behaviors are engaged in as a way to self-stimulate in the absence of enrichment in their environments, or to self-soothe in the absence of opportunities for comforting from other chimpanzees [6,9,12,22,23,24,25]. Both Poco and Safari were socially isolated for years before being rescued by the sanctuary. It is possible that they began poking, as well as self-clasping and rocking, as a way to self-stimulate or -soothe. Now, even though they live in a large social group (21 other chimpanzees), and were observed on at least one occasion receiving grooming from a companion, the ritualistically performed poking may be an obsessive compulsive behavior that still provides them with comfort. Others have suggested that these stereotypical behaviors may become detached from the original stimuli that brought them about [16]. This might explain why both individuals spent such a large percentage of the observation time engaging in these behaviors, even while performing other normal chimpanzee behaviors, such as getting groomed.

Self-injurious behaviors in primates are reported more often in individuals with negative early-life social experiences and in individuals exposed to a repeated number of moderately stressful events [24]. Both of these scenarios characterize the situations Poco and Safari lived in prior to being rescued by the sanctuary. Being separated from their mothers and families at an early age and living for prolonged periods of time socially isolated and in constant interaction with un-nurturing humans may have resulted in severe psychological distress that even years of living in a sanctuary may not heal. Previous research has found that biting in captive-living monkeys is commonly directed to body sites associated with acupuncture analgesia [24]. It is possible that Poco and Safari poke in areas that result in a similar outcome as a way to reduce their psychological distress. The finding that they both spent a large proportion of the observation time engaging in this stereotypical behavior is similar to what has been found in research in humans exhibiting symptoms of CPTSD. In addition to chronic affect dysregulation, aggression against the self is very common [26,36,53]. 

A criticism of previous studies in this area has been that using criteria that were developed in humans to diagnose chimpanzees with psychopathologies is not a powerful enough diagnostic tool, and suggested that using subjective secondary reports has methodological limitations [43]. Instead, it has been argued that assessing psychological distress in chimpanzees should be based upon objective species-specific behavioral criteria that are easily observable. The mixed methodology used in the current study—which included conducting interviews with caretakers and direct observations of normal and abnormal chimpanzee behavior—is a more powerful diagnostic tool and one way of validating that chimpanzees who are said to exhibit PTSD- and CPTSD-like symptoms actually display them. 

Most humans with PTSD are treated with cognitive behavioral therapy, exposure-based therapy, psychoanalysis, and/or pharmacological intervention (e.g., Selective Serotonin Reuptake Inhibitors) [53,54,55,56,57,58]. Additional treatment for CPTSD involves restoration of trust in other people, and control and power for the victim since the cause of the trauma is related to a dependence on a person in power who undermines the victim’s sense of agency [59,60]. 

Possible treatments for chimpanzees who exhibit symptoms of PTSD and CPTSD include restoring their sense of empowerment by giving them opportunities for decision making, such as deciding when to go inside and outside or choosing what to eat, providing opportunities for social interactions and allowing them to decide whom to interact with and when, as well as providing responsive veterinary care [12]. Almost all of the suggested forms of treatment are strategies implemented in various degrees by the Sweetwaters sanctuary. Additional treatments for self-injurious and stereotypical behaviors in chimpanzees include pharmacological interventions, and positive reinforcement training [11]. Given that only a small percentage of chimpanzees we initially surveyed the caregivers about met the criteria for PTSD or CPTSD, even if they did engage in at least one abnormal behavior, we agree with previous researchers that sanctuaries with enriched, social living arrangements and skilled, nurturing personnel are an effective form of treatment for most chimpanzees who experience disruptive maternal care and/or physical and psychological trauma [20]. 

## 4. Conclusions

The illegal hunting of chimpanzees for bushmeat in Africa is troublesome for many reasons, including the fate to which it often dooms the orphans of adults who are killed. The case studies of Poco and Safari demonstrate that as a result of maternal deprivation and isolation from conspecifics, some orphaned chimpanzees develop a variety of abnormal, stereotypical behaviors, and symptoms of psychological distress that persist many years after being rescued by sanctuaries and reintegrated into chimpanzee social groups. Their cases highlight some devastating life-long consequences of keeping chimpanzees as “pets”. While this remains a problem in Africa, reports estimate that as many as 700 chimpanzees are currently being kept as pets in the United States as well, though efforts to curb the practice are underway (c.f. S. 1324/H.R. 4306 Captive Primate Safety Act) [61]. 

This paper illustrates that, although accredited sanctuaries help facilitate the healing process for abused chimpanzees, sometimes the trauma endured may be too severe to fully recover from. 
